# Assessment of *Iris albicans* lange as potential antimicrobial and analgesic agent

**DOI:** 10.1371/journal.pone.0280127

**Published:** 2023-01-06

**Authors:** Muhammad Bilal, Attiqa Naz, Amjad Khan, Salman, Rukhsana Ghaffar, Azra Abrar

**Affiliations:** 1 Department of Pharmacy, Abasyn University, Peshawar, Pakistan; 2 Department of Pharmacy, Kohat University of Science and Technology, Kohat, Pakistan; 3 Department of Pharmacy, University of Malakand, Dir, Pakistan; 4 IBMS, Khyber Medical University, Peshawar, Pakistan; University of Arizona College of Medicine, UNITED STATES

## Abstract

Objective of the present work was to evaluate the presence of phytochemical constituents and pharmacological activities (antimicrobial and anti-nociceptive) of crude extract isolated from *Iris albicans* and its corresponding fractions. Extraction was made with methanol and extract was evaluated for the presence of different bioactive constituents, as per standard protocols. Extract and its corresponding fractions were evaluated for their antimicrobial and anti-nociceptive potential. Well diffusion method was used to evaluate the antibacterial effects while anti-nociceptive effect was studied using *in-vivo* models (acetic acid induced writhing, hotplate and tail immersion tests) at different dose level (100, 200 and 300 mg/kg of body weight) and compared with diclofenac sodium (dose = 10 mg/kg body weight). Results showed that the *I*. *albicans* extracts contained secondary metabolites including alkaloids, phytosterols, flavonoids, saponins, terpenoids, tannins, phenols, steroids, fixed oil, glycosides and carbohydrates. Furthermore, it was observed that different fractions in decreasing polarity order such as chloroform >*n*-Hexane > Ethyl acetate > Crude Methanol > Aqueous extract exhibited effective antimicrobial response against all test organisms. Results of the study showed that the extracts have significant antimicrobial and analgesic activity, providing evidence for its folklore use.

## Introduction

Antibiotics resistance is a global issue which lead to severe health hazards and compelling the researchers to either bring structural modification in the existing antibiotics or to develop natural alternative antimicrobial products [1]. Several species of ornamental plants have been considered for their tremendous antimicrobial activity [2, 3]. In South Asia, above 80% of the population depends on traditional medicines and local healers as the primary source of treatment [4]. This is mainly due to easy accessibility and affordability of consulting with traditional medical practitioners [4, 5]. In Pakistan, herbal drugs have been traditionally used for the treatment of pain, inflammation and infectious diseases for many decades [3]. However thorough evaluation is needed to scientifically prove their safety and efficacy.

*Iris albicans* Lange, belongs to *Iridaceae* family grows widely and distributed to Europe, Asia, North Africa, North America, Turkey and Yemen [6, 7]. It is a sterile, natural hybrid, and cannot produce seeds, spreads by rhizomal growth and division. Genu*s Iris* belongs to the *Iradaceae* family consist about 300 species of flowering plants. The genus also comprises of 46 taxa [8]. The genus *Iris* is widely known as rhizomatous herb. Due to high medicinal value of the genus *Iris*, it is used in the ailments like inflammation, liver dysfunction, cancer, bacteriological and virological infections [8]. This genus is also used in some other diseases like heart diseases, respiratory system disorders, constipation, sores, pimples, worms, hepatitis, and tuberculosis as immunomodulatory and also as anti-plasmodia infestations [8]. It is indigenous to Yemen and Saudi Arabia and in Pakistan, it is found in areas near Afghanistan borders (Bajauar Agency, Mohmand Agency). Globally known as white flag *Iris*, white cemetery *Iris* and cemetery *Iris* while locally known as “Gul-e-Zambak” (in Urdu and Pashto). It is a small plant and has a lot of uses in folk medicine. In Yemen and Saudi Arabia, it is utilized as main ingredient in cosmetic and deodorant industries [9]. Traditional system of medicine showed its utilization in rheumatism and as anti-gout agent in which paste of rhizomes of *I*. *albicans* are used [9].

In the present study, anti-nociceptive effect and anti-microbial potential (anti-bacterial and anti-fungal) of *I*. *albicans* has been evaluated. Plant extract was tested for the presence of different bioactive phyto-constituents and biological activities were evaluated for plant extract and its sub fractions as per standard protocols.

## Materials and methods

### Solvents and reagents

Solvents and chemicals used in this study were obtained from MERCK, Pakistan. Diclofenac sodium (Wellmark Pharma, Islamabad, Pakistan) was used positive control. The potato dextrose and nutrient agar were purchased from local market of Peshawar, Pakistan. Different antibiotic like levofloxacin, meropenem, fluconazole and nystatin were used as the reference antimicrobial agents and were of the analytical grade.

### Experimental animal models

Swiss albino mice (weighing 25–29 g of either sex) were obtained from Veterinary Research Institute (VRI) Peshawar, Pakistan, and all experiments were carried out with these animals in triplicates. Age of the mice was in the range of 5–6 weeks and their sex differences were not identified. Animals were kept under standard environmental conditions (temperature; 25 ± 0.5°C, dark/light cycles of 12/12 h, humidity; 50 ± 5%), with access to food and water. The protocols for animal handling and utilization in experiments were approved by the “Committee for Research Ethics” Department of Pharmacy, Abasyn University, Peshawar, Pakistan.

### Collection of the plant materials

Whole plant of the *Iris albicans* including leaves, stem and flowers were collected during the month of February and March from plain valley of District Bajaur (formerly known as Bajuar Agency), Pakistan. Plants were identified by the experts from Department of Botany, University of Peshawar, Peshawar, Pakistan and a verification code (Bot. 20151 (Pup)) was allotted.

### Extraction of the plant material

The aerial parts of the *I*. *albicans* were cleaned and dried, freed from rhizomes. Then the plant was chopped (5.57 Kg) and shade dried at room temperature for one month. The dried plant (3.20 Kg) was crushed to coarse powder (3.08 Kg), soaked in methanol (15 L) for two weeks at room temperature and filtered. This process was repeated three times. The extract was concentrated through rotary evaporator at 40 ± 2°C. For complete removal of methanol dried extract was kept at 40°Ć on water bath until the extract was completely dried. The dried crude extract was then subjected to fractionation [10, 11].

### Fractionation of crude extract

Sequential fractionation of the crude extract was performed using solvents of different polarity [12]. Fraction was started with solvents of low polarity and ended with highest polarity i.e. *n*-hexane > chloroform > ethyl acetate>aqueous. The isolated fractions were concentrated by rotary evaporation. The concentrated fractions were dried completely by evaporation of organic solvents on water bath at 40 ± 5°C and water was evaporated by keeping the concentrated fraction in hot air drier. The dried fractions were kept in a tightly closed container, properly labelled and reconstituted with different solvent on need basis for different experiments.

### Dose selection and grouping

For determination of anti-nociceptive activity, animals were distributed in five groups, each comprising of six animals (n = 6). Animals with similar age were placed in same group and age of treatment and control group were kept similar i.e., animal with a similar age were kept in same group. Normal saline was used as negative control while diclofenac sodium (Wellmark Pharma, Pakistan; 10 mg/kg of body weight; administered by intra peritoneal route) was used as positive control [13]. Animal groups were treated with 100 mg/kg body weight, 200 mg/kg body weight and 300 mg/kg body weight doses of extract and solvent fractions. Doses were selected on the basis of reported literature [13] to find out response at different level of dose.

### Phytochemical screening

Crude extract of *iris albicans* and its corresponding fractions (IACCF) were evaluated for their phytochemical constituents using standard protocols [13–15]. These protocols and procedure were related to identification of phyto-constituents like phenolic compounds, glycosides, alkaloids, saponins, terpenoids, flavonoids, anthraquinones, steroids and tannins as described [14, 15].

#### Test for carbohydrates

Extract was soaked in purified water (5 mL) for 5 min and filtered. Alcoholic α-naphthol solution (3 drops) was added to filtrate, taken in a test tube and observed the junction. Presence of carbohydrates was confirmed by appearance of violet ring at the junction [14, 15].

#### Test for alkaloids

Presence of alkaloids in the eaxtract was confirmed by Mayer’s test. Extract (0.5 g) was extracted with methanol and added HCl (2N). Heated the mixture at 40 ± 3°C, cooled and filtered it. This filtrate was reacted with Mayer’s reagent (potassium mercuric iodide) and checked the precipitate growth. Appearance of yellow color precipitate confirmed alkaloids presence [14, 15].

#### Test for saponins

Extract was soaked in boiling water in a test tube. After specified time, mixture was cooled and vigorously shaken till froth formation. Afterward the test tube was placed in stand for ~ 15 min and noted the results. Strongly positive (+++) meant more than 5 cm froth, (++) meant more than 2 cm froth, (+) meant <1 cm froth and (-) represent no froth [14, 15].

#### Test for phenols

The extract was soaked in water and filtered. Filtrate was reacted with ferric chloride solution (3–4 drops) and observed formation of precipitate. The appearance of bluish black color precipitate indicated presence of phenol [14, 15].

#### Test for glycosides

Presence of glycosides in extract was evaluated by Keller-kiliani test [2]. Extract (2 mL) was reacted with glacial acetic acid (1 mL) and FeCl_3_ (1–2 drops) than treated with concentrated H_2_SO_4_ (1 mL). Development of green blue color indicated presence of cardiac glycosides and vice versa [14, 15].

### Anti-microbial assay

Anti-microbial assay involved determination of both anti-bacterial and anti-fungal activity. Both the assays were performed by well diffusion method [16], using dimethyl sulphoxide (DMSO) as solvent and agar was used as growth media. Bacterial and fungal strains were obtained from Department of Microbiology, Abasyn University Peshawar, Pakistan. Expert from department of Microbiology confirmed their identity and purity as per USP-38/NF-33 protocols [17]. All the strains were kept in air tight containers, under standard conditions of storage as specified by USP 38–NF 33. Media was evaluated for its suitability for the growth of the selected strains of bacteria and fungi as per protocols for anti-microbial assay of United States Pharmacopeia [17]. During determination of suitability of media for microbial growth, different parameters for incubation like incubation time and temperature, were also optimized. The selected. Growth media was taken in plates and inoculated with selected strains of bacteria (*P*.*aeruginosa*, *E*.*coli*, *S*. *typhi*, *S*. *epidermis*, *S*. *aureus*, *C*. *freundii*, *A*. *israelii*, *Y*. *enterocolitis*.) and fungi (*A*. *nigre*, *A*. *flavus*, *P*. *notatum*), separately. Sterile borer (diameter = 6 mm) were used to make wells in the developed agar plates. Solution of test extract (100 mg/mL) was prepared in DMSO and each test extract (10 μL) was added in to separate well (1 mg / well). Meropenem (10 μg/disc) and levofloxacine (5 μg/disc) acted as positive control for bacterial culture plates while Nystatin (100 units/disc) and fluconazole (25 μg/disc) were used as positive control for fungal culture plates. In both the cases DMSO was used as negative control and its final volume was 100 μL. Bacterial culture plates were incubated at 37 ± 2°C for 24 h while fungi culture plates were incubated at 24 ± 2°C for 48–72 h. After completion of incubation period, zone of inhibition was measured around each well [18] from bottom of the plate.

### Anti-nociceptive activity

Anti-nociceptive activity of the extracts was evaluated by;

- Acetic acid induced writhes test
- Hot plate method
- Tail immersion method

#### Acetic acid induced writhes test

During the test, pain was initiated by intra peritoneal administration of acetic acid and treated with diclofenac sodium (10 mg/kg body weight; reference drug / positive control) and different doses (100, 200 and 300 mg/kg body weight) of test extracts from *I*. *albicans*. Crude extract and its fractions were administered orally by a gastric tube, about 30 minutes prior to initiation of experiment. Acetic acid solution (0.7%) was administered by intra peritoneal route (10 mL/kg body weight) to initiate pain (writhing response/count) in test animals. Then after 1 h, writhing initiated by the acetic acid were counted (for 30 min), after 5 min of latency period. The mice were treated with diclofenac sodium (standard drug/ positive control), normal saline (negative control; 10 mL/Kg body weight) and different doses of test extracts of *I*. *albicans* (100, 200 and 300 mg/kg body weight). Normal saline was used as solvent for preparation of solutions of plant extracts and dilution of diclofenac sodium injection. Oral administration of extracts and positive control was carried out through gastric tube and animals were properly acclimated to the procedure. Normal saline was used as negative control and was administered orally. The test animals were placed under constant surveillance. The total numbers of writhing were counted for 20 min. The percentage of anti-nociceptive effects was ascertained by following equation as described [18, 19].


$$\%\;Inhibition=\frac{No.of\;Control\;Writhes-No.of\;test\;Writhes}{No.of\;Control\;Writhes}x\;100$$ (1)

#### Hot plate method

The design of hot-plate method was described [20, 21] with slight changes in the context of present study. Mice were restrained from food overnight with readily access to water. Apparatus used resembled UGO hot-plate BASILE 7280, Germany, with some modifications, employed for analgesic activity of diclofenac sodium (10 mL/Kg body) and the IACCF. Hot-plate temperature was kept at 55 ± 0.5°C and mice were placed on the hot-plate. Mice response to radiant heat induced analgesia stimulus and paws licking or jumping were noted. These parameters were used as an indication or mice’s latency of analgesia responses. Paws licking or jumping time for each mouse was noted (reaction time) and 45 s was set as cut-off period avoiding damage to the limbs and paws. Only animals were selected showing jumping or licking reactions within the range of 15 s for current study (24 h) preceding to the initiation of experiment. Mice function as their own control as well as response time was calculated in triplicate preceding treatment; by 1 h interval. Each of the mice in the group was thereafter treated with a solution of IACCF (dose 100 mg/Kg, 200 mg/Kg and 300 mg/Kg) of each of the five extracts was prepared in normal saline.

- Test—1: solution of IACCF (dose- 100 mg/kg, 200 mg/kg, 300 mg/kg) of each of the five extracts,
- Test—2: A solution of Diclofenac Sodium (10 mL/Kg of body weight).

The reaction times of all mice were individually re-evaluated after 0, 30, 60, 90, 120 and 180 min of treatment. At last each treatment group’s test mean was calculated. Following treatment response or reaction time was represented by this mean (Ta) as well as specified percentage of thermal stimulus of pain by utilizing the below mentioned formula;

$$Thermal\;Stimulus\;percent\;of\;Protection=\frac{Mean\;of\;Test\;(Ta)-Mean\;of\;Control\;(Tb)}{Mean\;of\;Control\;(Tb)}$$ (2)

#### Tail immersion method

The lower 3 cm part of mice tail was appropriately marked and immersed in water bath at 55 ± 1°C. After a few seconds, the mice responded by flicking the tail. It is the reaction time (in 0.5–2 s units by a stopwatch). After each value the tail was dried carefully. For the test and standard drugs given orally reaction time was recorded prior and after administration periodically and 15 second was set up the cut off time. Test animals were adults Swiss albino mice. Test animals weighing between 25–29 g were utilized for tail immersion activity. Albino mice were scheduled in six groups. A solution of *I*. *albican* (dose-100 mg/kg/10 μL, 200 mg/kg/10 μL, 300 mg/kg/10 μL) of each of the five extracts was formed in N/S. Test—1: solution of *I*. *albican* (1-dose-100 mg/Kg.bw/10 μL, 200 mg/kg/10 μL, 300 mg/kg/10 μL) of each of the five extracts, Test—2: A solution of diclofenac sodium (10 mg/Kg of body weight) was prepared. After 60 min of oral administration of test and standard drugs, the observations were recorded in the interval of 0, 30, 60, 90, 120 and 180 min [19]. Like hot-plate method thermal pain stimulus calculated by following formula:

$$Thermal\;Stimulus\;percent\;of\;Protection=\frac{Mean\;of\;Test\;(Ta)-Mean\;of\;Control\;(Tb)}{Mean\;of\;Control\;(Tb)}$$ (3)

#### Statistical analysis

The results were analyzed by statistical means utilizing one-way ANOVA followed by Dunnet’s t-test, using the software “Graph Pad Prism” version 5 (San Diego, CA, USA). *P (0*.*05)* was considered as significant value. Results were presented as Mean ± SEM.

## Results

### Phytochemical screening of *Iris albicans*

In the current study, methanolic extract of *I*. *albicans* and its corresponding fractions were screened for the presence of secondary metabolites like flavonoids, glycosides, terpenoids, alkaloids, steroids, saponins and reducing sugar. Methanolic extract of *I*. *albicans* and its corresponding fractions displayed the presence of all active metabolites as shown in Table 1, It confirms importance of the extract from *I*. *albicans* with respect to the presence of medicinally important constituents.

**Table 1:** Phytochemical analysis of crude extract of *I*. *albicans*.

Bioactive Agents (metabolites)		Crude Extract
Alkaloids		+
Saponins		+
Terpenoids		+
Tannins		+
Flavonoids		+
Phenols		+
Steroids		+
Fixed oil		+
Glycosides		+
Carbohydrates	Fehling test	+
	Molisch test	+
Proteins	Millon test	-
	Ninhydrin test	-

+ = Presence; Means that the test shows positive result for the presence of particular phytochemical

- = Absent; Means that the test showed negative result and the particular phytochemical has not been detected in sample

### Biological evaluation of *I*. *albicans*

Crude extract of *I*. *albicans* and its corresponding fractions were evaluated for antibacterial, antifungal assay through standard protocols.

#### Evaluation of anti-bacterial activity

The *I*. *albicans* and its corresponding fractions were evaluated through well diffusion method against eight bacterial strains (*staphylococcus aureus*, *escherichia coli*, *salmonella typhi*, *staphylococcus epidermidis*, *pseudomonas aeruginosa*, *citrobacter freundii*, *actinomycetes israelii* and *yersinia enterocolitica)*. Mereopenem and levofloxacin were used as standard (reference) drugs. All samples were effective against *pseudomonas aeruginosa* while comparing with the standard levofloxacin, ethyl acetate fraction showed moderate activity against all test organisms except *staphylococcus epidermidis*. The results obtained for samples against *citrobacter freundi* and *actinomycetes israelii* were almost same and significant compare to both standards used while zone of inhibition of samples against *salmonella typhi* and *staphylococcus aureus* were same and moderately effective. Results of antibacterial activity of *I*. *albicans* extract and its sub fractions are presented in Table 2.

**Table 2:** Antibacterial activity of *I*. *albicans* crude and corresponding fractions.

Bacterial strains	Zone of inhibition (mm) against test organisms							
	Crude	*n*- hexane	CHCl_3_	Et-Ac	Aq	DMSO	MEP	LEVO
*P*. *aeruginosa*	19	15	17	16	25	-	30	6
*E*. *coli*	17	12	12	10	12	-	12	11
*Salmonella*	13	13	14	13	10	-	28	21
*S*. *epidermidis*	10	10	11	14	10	-	18	16
*S*. *aureus*	16	13	12	13	12	-	17	17
*C*. *freundii*	12	22	16	12	15	-	21	23
*A*. *israelii*	11	10	13	12	10	-	20	19
*Y*. *enterocolitica*	15	16	18	20	15	-	30	32

Data shows zone of inhibition (mm), presented as mean of three determinations (n = 3)

- shows no zone of inhibition

Et-Ac = Ethyl acetate; CHCl3 = Chloroform; Aq = Aqueous; DMSO = Dimethyl sulphoxide; MEP = Meropenem; LEVO = Levofloxacin

**Fig 1:**
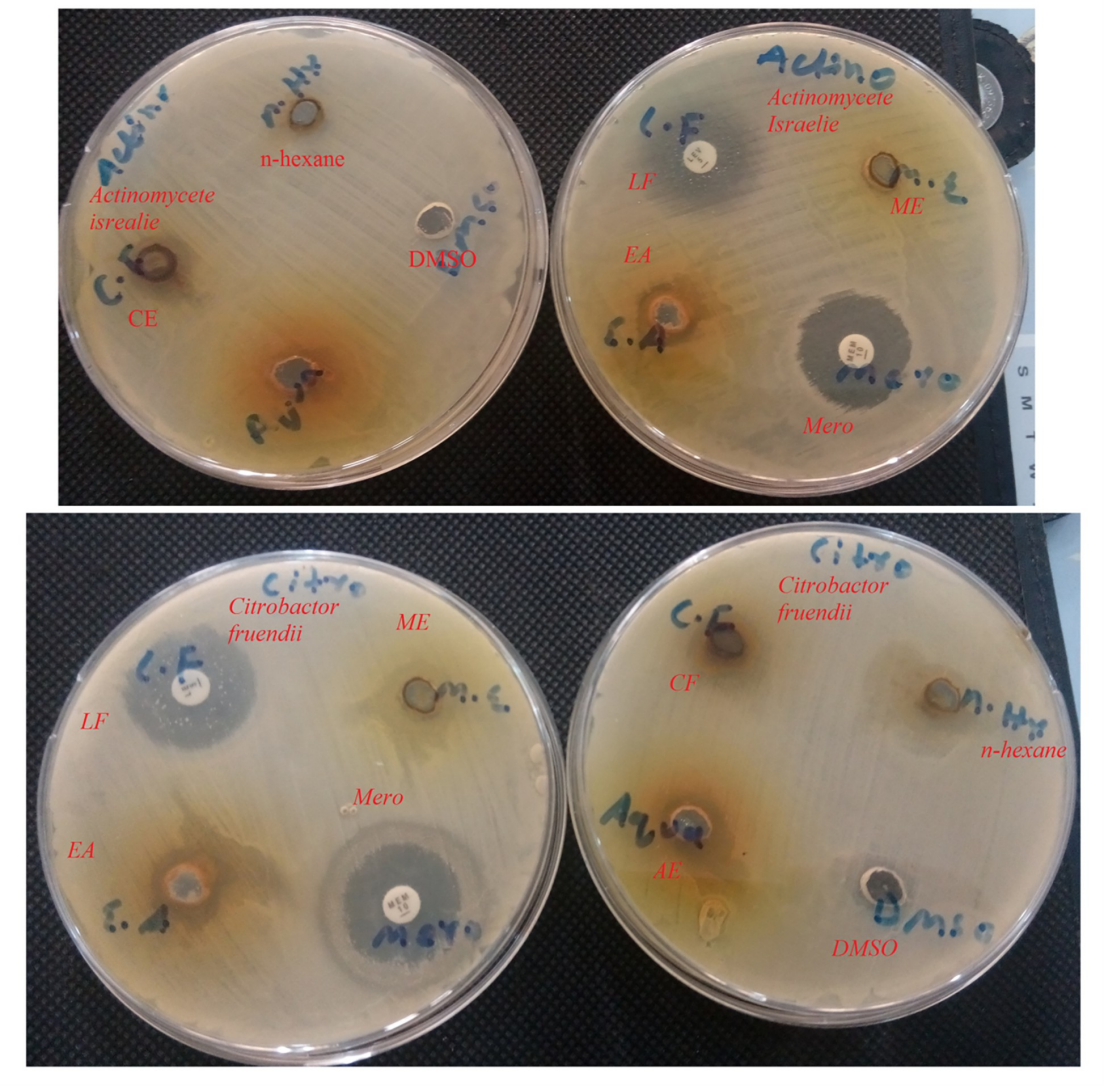
Zone of inhibition of extract against specific bacteria. Aqua: Aqueous Sub-fraction: C.F: Chloroform Sub-fraction; DMSO: Dimethyl Sulfoxide Sub-fraction; E.A: Ethyl Acetate Sub-fraction; L.F: Levofloxacin; M.E: Methanolic Extract; MERO: Meropenam.

**Fig 2:**
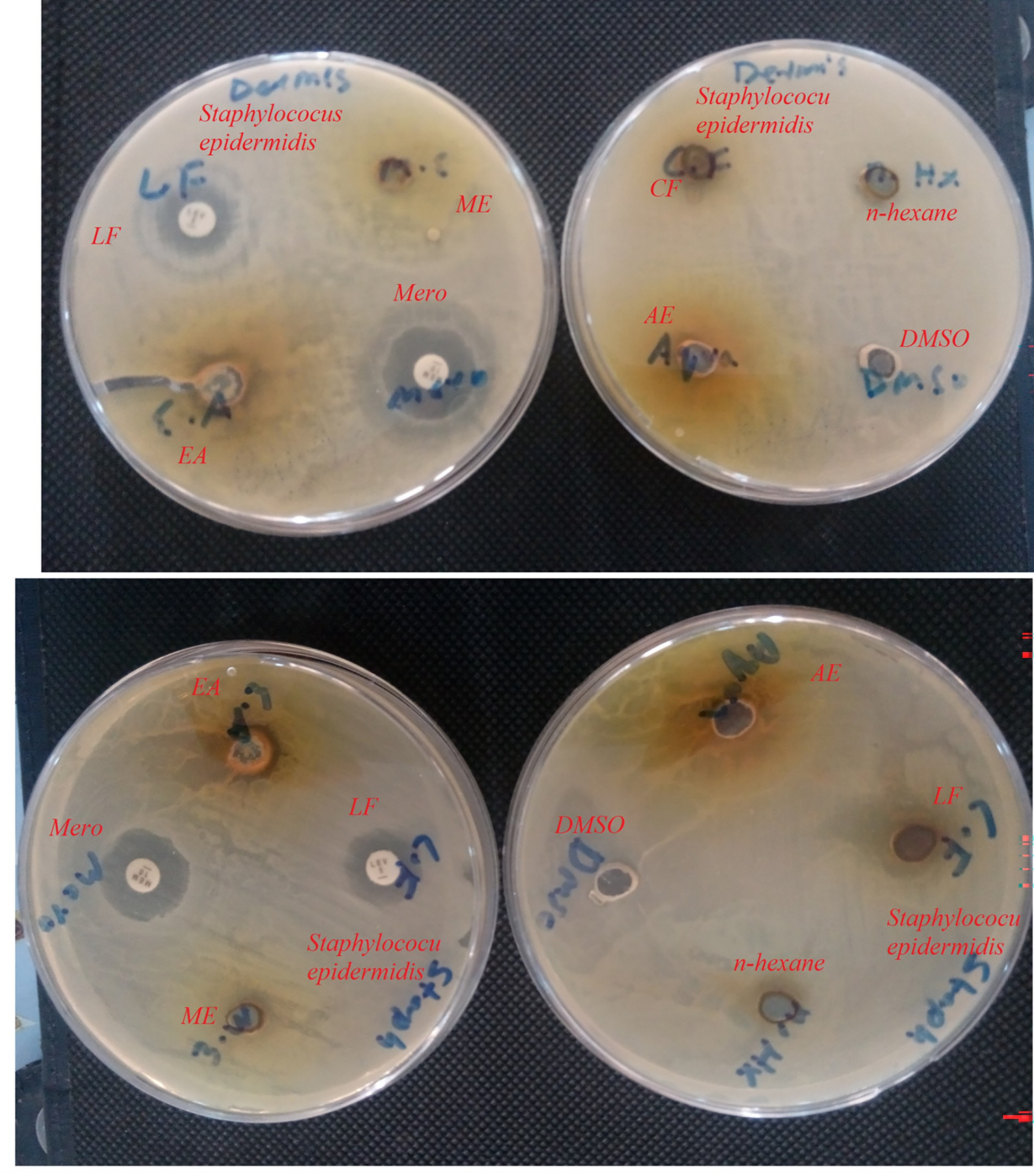
Inhibition of bacterial growth by extract. Aqua: Aqueous Sub-fraction: C.F: Chloroform Sub-fraction; DMSO: Dimethyl Sulfoxide Sub-fraction; E.A: Ethyl Acetate Sub-fraction; L.F: Levofloxacin; M.E: Methanolic Extract; MERO: Meropenam.

Figs 1 and 2 represents zone of inhibition of extracts against specific bacteria.

#### Anti-fungal activity of *I*. *albicans*

The crude and fractions were subjected to antifungal assay using nystatin and fluconazole as standard drugs. Fungal strains used in the study included *aspergillus niger*, *aspergillus flavus* and *penicillium*. The crude and corresponding fractions were effective against *A*. *niger* while DMSO was used as solvent and was inactive against all fungal strain as shown in Table 3.

**Table 3:** Antifungal activity of *I*. *albicans* crude and corresponding fractions.

Fungal strains	Zone of inhibition (mm) against test organisms							
	Crude	*n*- hexane	CHCl_3_	Et-Ac	Aq	DMSO	NYS	FLUC
*A*. *niger*	10	11	15	17	12	-	15	12
*A*. *flavus*	14	10	13	15	11	-	10	13
*P*. *notatum*	11	11	13	16	11	-	16	10

Data is presented as mean value of three determinations

Et-Ac = Ethyl acetate sub-fraction; CHCl_3_ = Chloroform sub-fraction; Aq = Aqueous sub-fraction; DMSO = Dimethyl sulphoxide sub-fraction; NYS = Nystatin sub-fraction; FLUC = Fluconazole sub-fraction

**Fig 3:**
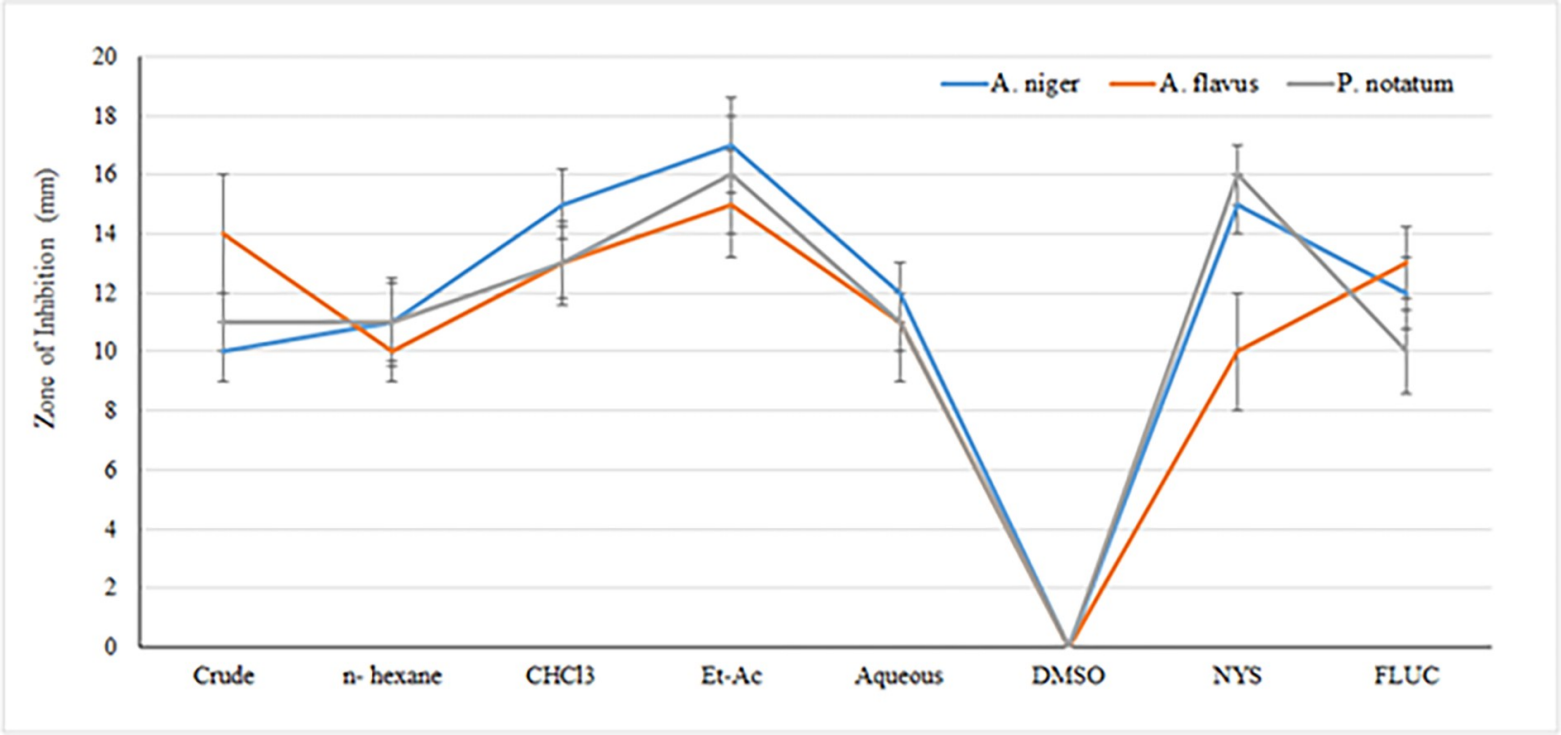
Comparison of antifungal activity of *Iris albicans* crude extract and its corresponding fractions.

Comparison of anti-fungal effect produced by the crude extracts and its sub fractions is presented in Fig 3.

### *In vivo* biological activity of *I*. *albicans*

#### Analgesic activity

*I*. *albicans* has been traditionally used for their analgesic and anti-gout effect which makes basis for evaluation of its anti-nociceptive potential. Analgesic activity was based on acetic acid induced writhe’s test, hot-plate test and Tail-immersion test.

*Acetic acid induced writhing test*. The effect of *I*. *albicans* and its corresponding fractions on the writhing response in mice is shown in Table 4. The plant extract and its fractions numerically (*P = 0*.*05*) prevented the acetic acid induced effects and highest inhibitory response (41.72 to 60.51 as compared to control) was observed at highest dose (300 mg/kg body weight). The inhibitory potential of *I*. *albicans* and its corresponding fractions at 300 mg/kg was better but in some cases was lower than diclofenac sodium at a dose of 10 mg/Kg (62.8%). All fractions displayed analgesic activity but chloroform and n-hexane showed significant inhibition. In the pursuing of acetic acid test the extracts of s *I*. *albicans* and its corresponding fractions showed numerically significant activity. The order of inhibition is;

Chloroform>n-Hexane**>**Ethyl acetate**>**Crude Methanol**>**Aqueous.

**Table 4:** Effect of *IACCF* on acetic acid-induced abdominal constriction in mice.

Samples	Dose/kg	Inhibitory Response	% inhibition
Negative Control	10 mL	32.2±1.41	-
Diclofenac (Positive Control)	0.25 mg	19.8 ±2.31	21.8%
Methanolic	100 mg	24.0±1.81	19.2%
	200 mg	21.4±1.60	20.1%
	300 mg	13.2±1.98	20.3%
n-Hexane	100 mg	28.8±2.22	19.5%
	200 mg	23.2±1.15	21.2%
	300 mg	14.6±1.86	26.3%
Chloroform	100 mg	24.0±0.94	16.2%
	200 mg	19.4±0.50	20%
	300 mg	16.4±0.81	21.2%
Ethyl Acetate	100 mg	27.8±1.20	16.4%
	200 mg	23.4±1.32	18.2%
	300 mg	19.8±1.22	20.1%
Aqueous	100 mg	28.8±1.06	15.5%
	200 mg	23.8±1.41	17.2%
	300 mg	20.2±1.56	20%

Data is presented as Mean ±SEM

Percent inhibition was calculated on the basis of mean values

It was found that less hydrophilic extracts, showed better analgesic activity in comparison with more hydrophilic fractions. Higher dose (300 mg/kg) of all extracts showed better inhibition of writhes in mice while lower doses (200 and 100 mg/kg) of all extract fractions, except chloroform and n-hexane displayed moderate effects.

*Hot-plate test in mice*. The findings of the hot-plate test showed that the *IACCF* has potential anti-nociceptive effect in relation to control groups. The *IACCF* in doses of 100, 200 and 300 mg/kg of body weight were effective and comparable with diclofenac sodium injection, (10 mg/kg), as shown in Table 5. No adverse effects seen with *I*. *albicans* extract and no animals died of *IACCF* treatment.

**Table 5:** Effect of *Iris albicans* crude and its corresponding fractions on hot-plate test in mice.

Samples/ Extract	Dose/kg	Reaction time in minutes					
		0 min	30 min	60 min	90 min	120 min	180 min
Saline	10 μL	4.2 ± 0.11	4.3±0.37	5.9±0.30	5.8±0.37	5.9±0.30	5.2±0.42
Diclofenac	10 mg	4 ± 0.26	8.5±0.42	8.6±0.67	8.6±0.67	8.5±0.66	8.4±0.42
Methanolic	100 mg	3.4 ± 0.14	5.8±0.30	5.9±0.30	7.3±0.42	7.3±0.42	7.2±0.21
	200 mg	4.4 ± 0.19	6.5±0.22	7.2±0.36	7.8±0.30	7.8±0.47	7.7±0.47
	300 mg	3.7 ± 0.23	7.3±0.33	7.7±0.42	8.0±0.36	8.0±0.51	7.9±0.47
n-hexane	100 mg	3.4 ± 0.26	6.3±0.49	6.9±0.30	7.4±0.47	7.5±0.42	7.4±0.56
	200 mg	4.1 ± 0.3	7.2±0.51	7.8±0.40	8.0±0.25	8.0±0.5	7.9±0.47
	300 mg	4.3 ± 0.28	8.3±0.30	8.4±0.91	8.5±0.61	8.4±0.71	8.3±0.22
Chloroform	100 mg	4.2 ± 0.35	6.5±0.5	7.0±0.36	7.1±0.47	7.4±0.42	7.6±0.21
	200 mg	3.1 ± 0.27	7.3±0.33	7.8±0.30	8.1±0.22	8±0.57	8±0.47
	300 mg	4 ± 0.16	8.4±0.42	8.5±0.76	8.5±0.42	8.4±0.76	8.4±0.47
Ethyl acetate	100 mg	3.5 ± 0.4	5.83±0.30	6.3±0.55	6.5±0.30	6.7±0.49	7.1±0.40
	200 mg	3.9 ± 0.17	6.0±0.36	7.0±0.42	7.3±0.33	7.3±0.42	7.6±0.49
	300 mg	3.7 ± 0.24	6.7±0.55	7.7±0.42	7.8±0.40	7.9±0.51	7.7±0.55
Aqueous	100 mg	4.1 ± 0.3	5.6±0.33	5.8±0.44	5.8±0.47	6.1±0.55	6.7±0.40
	200 mg	3.5 ± 0.28	6.5±0.42	6.7±0.25	6.3±0.42	7.1±0.49	7.0±0.40
	300 mg	4 ± 0.19	7.0±0.51	7.6±0.49	7.8±0.44	7.7±0.47	7.4±0.5

Data is presented as Mean ± SEM

**Fig 4:**
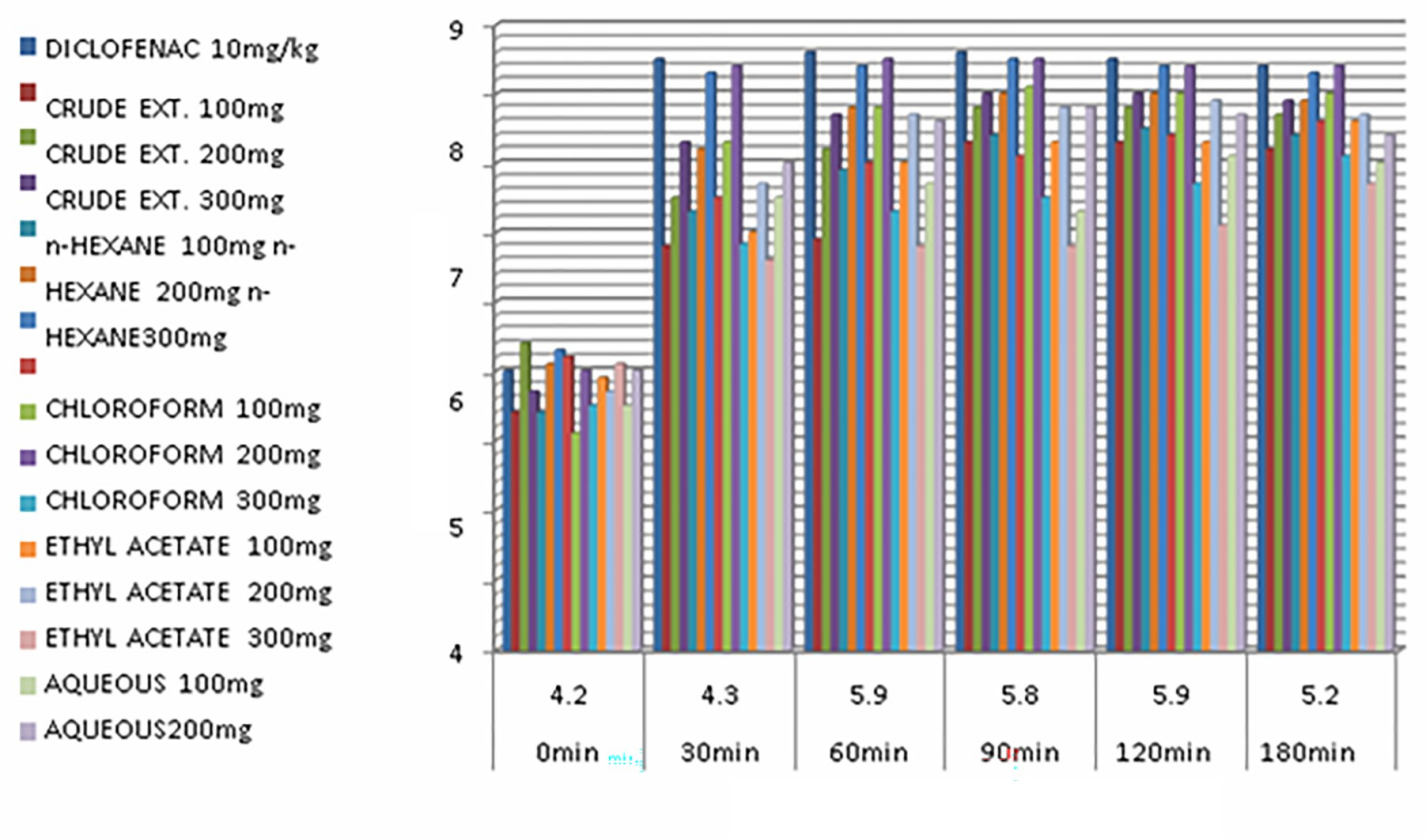
Analgesic activity of *Iris albicans* crude extract and its sub-fractions. Analgesic effect was evaluated using Hot -Plate test in animal model (n = 6).

Comparison of analgesic effect of the crude extract and its sub-fractions is presented in Fig 4.

*Tail-immersion test in mice*. In the tail immersion test all fractions displayed analgesic activity. However, chloroform and n-hexane showed maximum inhibition followed by ethyl acetate, crude and aqueous. In tail immersion test; the extracts of *iris albicans* showed numerically significant dose dependent activity i.e. 300 mg > 200 mg > 100 mg. It was also observed that lipid soluble extracts showed better activity as compared to the low lipid soluble extracts. At 300 mg/kg dose, all the extracts possessed excellent decrease in licking and jumping response in mice while 200 mg dose of all extracts except chloroform and n-hexane fractions; showed moderate effects. However; all extracts (100, 200 and 300 mg/Kg body weight) significantly increase latency period as shown in Table 6.

**Table 6:** Effect of *Iris albicans* crude and its corresponding fractions on tail-immersion test in mice.

Samples	Dose/Kg	Reaction time in Minutes					
		0 min	30 min	60 min	90 min	120 min	180 min
Saline	10 ml	0.82±0.14	0.81±0.16	0.9±0.13	0.84±0.20	0.83±0.19	0.87±0.20
Diclofenac	10 mg	0.84±0.11	2.9±0.25	3.3±0.33	3.42±0.27	4.8±0.17	4.8±0.18
Crude	100 mg	0.81±0.16	2.1±0.05	2.8±0.12	3.1±0.21	3.3±0.21	3.1±0.27
	200 mg	0.83±0.14	2.7±0.16	3.1±0.04	3.33±0.11	3.6±0.15	3.5±0.18
	300 mg	0.81±0.18	2.9±0.27	3.2±0.1	3.40±0.15	4.8±0.1	4.5**±**0.1
n-hexane	100 mg	0.81±0.16	2.3±.09	2.5±0.18	2.9±0.15	3.1±0.152	3.3±0.18
	200 mg	0.83±0.14	2.7±0.16	3.5±0.18	3.2±0.1	3.7±0.42	3.9±0.20
	300 mg	0.81±0.1	2.9±0.27	3.8±0.1	3.4±0.08	4.80±0.1	4.5**±**0.3
Chloroform	100 mg	0.81±0.16	2.8±0.12	3.1±0.1	3.2±0.18	3.7±0.1	3.6±0.2
	200 mg	0.83±0.14	3.1±0.2	3.3±0.18	3.4±0.18	4.5±0.3	4.4±0.17
	300 mg	0.81±0.18	3.4±0.1	3.7±0.15	3.7±0.18	5.1±0.18	4.9±0.1
Ethyl acetate	100 mg	0.81±0.16	2.4±0.14	3.1±0.16	3.2±0.16	3.7±0.19	3.6±0.19
	200 mg	0.83±0.14	2.6±0.21	3.2±0.04	3.4±0.18	4.3±0.19	4.4±0.13
	300 mg	0.81±0.18	2.8±0.20	3.3±0.16	3.4±0.19	4.6±0.06	4.5±0.19
Aqueous	100 mg	0.81±0.16	2.7±0.09	2.9±0.14	3.1±0.21	3.8±0.04	3.6±0.04
	200 mg	0.83±0.14	2.8±0.16	3.1±0.04	3.3±0.16	4.3±0.18	4.4±0.03
	300 mg	0.81±0.18	2.9±0.27	3.2±0.16	3.4±0.19	4.7±0.2	4.6±0.03

Data is presented as Mean ± SEM

## Discussion

The phytochemical analysis of *I*. *albicans* and its corresponding fractions showed the presence of different bioactive constituents (primary and secondary metabolites) including alkaloids, saponins, terpenoids, tannins, flavonoids, phenols, steroids, fixed oil, glycosides and carbohydrates. Presence of different bioactive constituents provides a scientific reason for the its flockier use.

*I*. *albicans* and its corresponding fractions have excellent antimicrobial effects against a wide range of microorganisms. *I*. *albicans* and its corresponding fractions showed excellent inhibition potential against bacterial and fungal species like *escherichia coli*, *staphylococcus aureus*, *staphylococcus epidermidis*, *pseudomonas aeruginosa*, *salmonella typhi*, *citrobacter freundii*, *actinomycetes israelii*, *yersin*i, *aspergillus niger*, *aspergillus flavus* and *penicillium*. These antimicrobial results were unique and novel due to employing of well diffusion technique for crude extract of *I*. *albicans* and its corresponding sub-fractions. Furthermore, globally accepted solvents have been used during the study [4, 22, 23] minimizing the interference by the solvents.

The antimicrobial studies revealed that *I*. *albicans* and its corresponding fractions have the potential for the isolation and discovery of new drug products from plant. Chloroform extract showed same inhibitory potential as levofloxacin (zone of inhibition 24 mm). The sequential antimicrobial potential efficacy is as;

Chloroform extract >*n*-hexane >methanol (crude) extract >ethyl acetate extract >aqueous extract

Comparison with reported literature showed that flavonoids, phenols, phtosterols and terpenoids were responsible for the antimicrobial activity of *I*. *albicans* and its corresponding fractions [2, 3, 9]. These compounds were copiously found in crude methanol, ethyl acetate as well as chloroform extracts. This study showed that *IA* it is a potential candidate for isolation of an effective anti-microbial drug.

The anti-nociceptive potential of *I*. *albicans* and its corresponding fractions were analyzed by;

- Acetic acid induced writhes test
- Hot plate method
- Tail immersion test

All the tests were performed according to the reported methods [24, 25] mice models. These analgesic models were chosen to confirm authenticate and validate the possessing of both anti-nociceptive responses i.e., central and peripheral responses, so as to allow the evaluation of the expected mechanism of effect of constituent of the extract [26].

Being an irritating agent; acetic acid induces pain, with characteristic abdominal constrictions by stimulating local receptors of peritoneum [25]. Abdominal constrictions effect has been linked with presence of prostanoids e.g. raised levels of PG-E and PGF2α in the fluids of peritoneal and lipoxygenase products. These effects had been reported after the administration of acetic acid [27]. The acetic acid-induced test is very sensitive, simple and capable of detecting analgesia at lowest possible dose which is not seen with other methods and test, like the tail flick method [28]. The inhibitory effect exhibited by the extracts is proposed to have a peripherally mediated analgesic activity which may be linked to inhibition of lipo-oxygenases and cyclooxygenases [25]. Peritoneal administration of dilute acetic acid increases release of mediators which in turn activate the terminals of primary afferent fibers, preventing nociception [29]. The multiple extracts caused a remarkable prolongation of response time to pain stimuli [30]. Centrally mediated action may likely be through opioid receptors in the CNS [30, 31]. Opioid analgesics relieve pain by raising the pain threshold at the spinal cord level and by altering the brain’s perception of pain. The ability of the extracts of *Iris albicans* to suppress pain perception in triplet (the acetic acid-induced test, the hot plate and tail immersion tests) suggests that the anti-nociceptive activity is possibly mediated by a series of mechanisms that comprises both central as well as peripheral pathways of pain intuition. Also glycosides, especially alcoholic glycosides, have proven analgesic and antimicrobial activities [3]. Constituents such as triterpenes and saponins produced anti-nociceptive actions on nervous system and cardio-vascular effects [32]. It may also contributed to the analgesic effect.

## Conclusion

It was concluded from the study that *I*. *albicans* has many medicinally important phytochemicals like alkaloids, flavonoids, phtosterols, phenols, carbohydrates, terpenoids, tannins, and saponins, and has shown significant pharmacological activities such as analgesic effect, antibacterial effect and anti-fungal activity. This study will provide a scientific base for its medicinal use. Furthermore, it is suggested that *I*. *albicans* should be further processed for isolation, characterization and structure elucidation of bioactive compounds, responsible for pharmacological activities.

### Limitations of the study

The study was conducted with objective to provide scientific proof for acute use of the extract from *I*. *albicans*. Some of the limitations of the study are;

- The study provides results for acute use of the I. albicans extract and no data has been provided for chronic use of the extract.
- Qualitative characterization of individual fraction was not performed.

### Future perspective

In the presented study only the acute effect of the plant extract has been studied. The effect produced by chronic use of the *IA* extract will be evaluated.
